# A Double-Blind, Placebo-Controlled Study on the Effects of Lutein and Zeaxanthin on Neural Processing Speed and Efficiency

**DOI:** 10.1371/journal.pone.0108178

**Published:** 2014-09-24

**Authors:** Emily R. Bovier, Lisa M. Renzi, Billy R. Hammond

**Affiliations:** 1 Vision Sciences and Human Biofactors Laboratories, Department of Psychology, The University of Georgia, Athens, Georgia, United States of America; 2 Abbott Nutrition, Columbus, Ohio, United States of America; Arizona State University, United States of America

## Abstract

Lutein and zeaxanthin are major carotenoids in the eye but are also found in post-receptoral visual pathways. It has been hypothesized that these pigments influence the processing of visual signals within and post-retina, and that increasing lutein and zeaxanthin levels within the visual system will lead to increased visual processing speeds. To test this, we measured macular pigment density (as a biomarker of lutein and zeaxanthin levels in brain), critical flicker fusion (CFF) thresholds, and visual motor reaction time in young healthy subjects (n = 92). Changes in these outcome variables were also assessed after four months of supplementation with either placebo (n = 10), zeaxanthin only (20 mg/day; n = 29) or a mixed formulation containing 26 mg/day zeaxanthin, 8 mg/day lutein, and 190 mg/day mixed omega-3 fatty acids (n = 25). Significant correlations were found between retinal lutein and zeaxanthin (macular pigment) and CFF thresholds (*p*<0.01) and visual motor performance (overall *p*<0.01). Supplementation with zeaxanthin and the mixed formulation (considered together) produced significant (*p*<0.01) increases in CFF thresholds (∼12%) and visual motor reaction time (∼10%) compared to placebo. In general, increasing macular pigment density through supplementation (average increase of about 0.09 log units) resulted in significant improvements in visual processing speed, even when testing young, healthy individuals who tend to be at peak efficiency.

## Introduction

Aging, even in the absence of overt neural pathology, is often accompanied by a decline in central nervous system efficiency that tends to manifest as deficits in higher-level executive functions such as short-term memory, judgment and decision-making ability, inhibition of pre-potent responses and working memory; and as deficits in more basic processing functions, such as integration of sensory and motor responses (e.g., reaction time). Of these deficits, a slowing of processing speed appears to be central and not as amenable to the same compensatory mechanisms available for other, less time-dependent, functions [1]. Numerous neurobiological systems have been identified to account for such slowing [2] but central to many is the idea of reduced efficiency [3]. An efficient nervous system likely reflects a careful balancing of speed and precision. Numerous studies have shown that declines in both speed and precision, which are often characterized as neural “noise” [4], occur significantly with age.

The above-described deficits seem to manifest at different times along the aging trajectory. Higher-level deficits, such as loss of short-term memory ability, tend to be easily detectable later in life, but younger individuals with varying degrees of cortical health and neural loss tend to perform relatively similarly on these tasks in absence of frank disease and are difficult to differentiate. More basic processing functions, however, such as ability to process movement of rapidly fluctuating stimuli, are highly variable across the lifespan, and differences are detectable even in younger adults who otherwise perform near ceiling on “higher” cognitive tasks [5]–[6]. Consequently, these tasks may be used to distinguish performance ability in younger and older adults alike (providing a tool for direct comparison across decades of life), and serve as methodology to easily assess biomarkers that could be used to characterize meaningful individual differences in brain function.

In the visual domain, for example, a large proportion of the brain is dedicated to lower and higher level processing of visual input. Unlike many complex cognitive tasks, visual stimuli can be reduced to their simplest form, allowing more simple neural systems to be invoked. For example, critical flicker thresholds are likely determined at the level of the visual cortex [7]. Using simple sensory stimuli as a means of studying more complex executive functions such as memory is well articulated as the common cause hypothesis, which argues that sensory (especially visual) and cognitive function are mutually reflective because they are both expressions of the same underlying physiological architecture [8]–[9]. Even the most complicated of brain functions are products of basic sensory input, and these measures correlate with other measures of cognitive function in older adults. Simple reaction time, for instance, changes predictably with age, and is impaired in individuals with neurodegenerative diseases [10]–[12] and with traumatic brain injury [13]–[14] and is a useful indicator of neurological health [15].

If processing speed is an important biomarker of optimal brain function and a predictor of future cognitive decline, it is also meaningful that processing speed correlates with other factors that are known to be modifiable. Individuals of all ages, and on a nearly daily basis, attempt to modify their own processing speed, mental alertness, and clarity. Pharmaceuticals like caffeine increase processing speed, but the effects tend to be transient (e.g., by blocking adenosine receptors) and habituate [16]–[17]. Such observations beg the question of whether there are less transient ways to increase processing speed and neural efficiency. For example, exercise appears to significantly increase executive functions, processing speed, and working memory in the elderly [18], and those effects tend to last longer and work via multiple mechanisms of action (e.g., by improving blood flow, reducing inflammation, increasing the supply of brain-derived neurotrophic factor, etc.).

Another approach to improving processing and preventing decline is dietary. The brain is, of course, composed of elements of the diet, and it is therefore reasonable to expect that the precise makeup of the diet and uptake of dietary components across the blood-brain barrier and into neural tissue could influence brain function. For example, over half of the brain is fat and, hence, whether the diet is composed of more rigid saturated fats or more fluid fats (like docosahexaenoic acid, DHA) influences brain fat composition and its resultant function [19]–[21]. When examining the other dietary components that make up the nervous system, one interesting component is the presence of dietary carotenoids lutein (L) and zeaxanthin (Z). These carotenoids are, perhaps, best known for their function in the neural retina, where they are found in high concentration and, along with their isomer meso-zeaxanthin, are termed macular pigment (MP). L and Z (as MP) are known to serve light-absorbing optical [22]–[23] functions, as well as antioxidant and anti-inflammatory functions [24]. L and Z are also present throughout human brain [25]–[27], even within the first year of life [28]. Studies using a monkey model have shown that measuring L and Z in the retina (macular pigment optical density, MPOD) represents a good proxy for the amount of L and Z in the brain [29]. MPOD has also been associated with visual processing speed [5], [30] and cognitive performance in both healthy elders and those with mild cognitive impairment [27], [31]–[33]. Consequently, MPOD may reflect dietary variables that change depending on an individual's cognitive status, and that correlate with other indices of brain integrity and cognitive health, such as processing speed.

This line of research is relatively new and whether changing neural L and Z levels can actually result in changes in measures like processing speed and cognitive function is not yet known. Only one supplementation trial has been conducted to test the effects of lutein supplementation on cognitive performance [31], which found that, when compared to placebo, a formulation combining L, Z and DHA resulted in cognitive improvements across a number of domains (short-term memory, verbal fluency, etc) in elderly women. In the present study, we used a similar design but focused on those measures known to distinguish even healthy younger subjects: visual processing speed and efficiency.

## Method

### Subjects and intervention

Subjects were young adults, aged 18–32 years, recruited from the University of Georgia and surrounding Athens community (N = 92; 36 males, 56 females; average age 21.7 years). Exclusion criteria included: corrected visual acuity worse than 20∶60 (Snellen notation), a history of ocular disease, and the use of a lutein or zeaxanthin supplements in the last six months. Sixty-four of the initial 92 participants participated in the intervention arm of the study. At the time of enrollment, subjects were randomly assigned to one of three treatment groups. Supplements were provided in an unmarked bottle with instructions for consumption listed under the cap, thus blinding the specific treatment received. Data collection and analysis was completed without knowledge of the subject's treatment group.

Subjects completed one of three different interventions, as follows: 29 participants (14 males, 15 females) received a supplement containing 20 mg Z/day (EyePromise Zeaxanthin, ZeaVision, LLC; Chesterfield, MO); 25 participants (9 males, 16 females) received a supplement containing 26 mg Z, 8 mg L, 190 mg mixed n-3 fatty acids/day (EyePromise vizual EDGE, ZeaVision, LLC; Chesterfield, MO), referred to as the “multi” condition. An additional 10 participants (4 males, 6 females) received a placebo. All interventions lasted for a four-month period. The remaining subjects either voluntarily dropped from the study (N = 8), were dropped due to noncompliance (e.g., 50% or less compliant according to pill counts half way through the intervention, N = 11), or were lost to follow-up (N = 9). The subjects that dropped were not different (as assessed by age, sex, and our inclusion/exclusion criteria) than those that remained within the study.

### Materials and Methods

All procedures were approved by the University of Georgia's Institutional Review Board and adhered to the principles in the Declaration of Helsinki. Subjects provided written consent to participate in the study. MPOD, temporal visual function and two additional tests of sensorimotor ability were completed at two time points within a one-week time frame, in order to establish a baseline. The average of those two visits was used for cross-sectional analyses. At the second baseline visit, subjects received a four month supply of supplements. Participants were instructed to take the supplements with a meal and were asked to refrain from making substantial changes to their diet during the course of the intervention. This was verified with bi-monthly checks and at the final laboratory visit. To promote compliance, phone calls were made to each subject every two weeks over the course of the four-month intervention, to gauge compliance and determine whether or not supplements were well tolerated. At the end of the four-month supplementation period, subjects completed MPOD, temporal visual and sensorimotor tests one final time.

#### Measuring MPOD

MPOD was assessed psychophysically using customized heterochromatic flicker photometry (cHFP). Measurements were conducted on a table-top device described by Wooten et al. [34]. cHFP requires the subject to adjust the intensity of a 460 nm light (maximally absorbed by MP), which alternates in square-wave at an individually-customized rate with a 570 nm light (outside of MP's absorbance spectrum), until the perception of flicker is eliminated. Measurements were taken in the fovea, where MP is present, and the parafovea, where MP is not detectable. The log-difference in the amount of energy required to eliminate the flicker between the fovea and the parafovea was used to derive MPOD at 30-minutes of retinal eccentricity.

#### Measuring temporal processing speed and sensorimotor ability

Critical flicker fusion (CFF) thresholds were measured using a customized, LED-driven tabletop device described by Wooten et al. [35]. The test stimulus consisted of a 1-degree 660 nm target at the center of a 5.5-degree 660 nm surround, separated by a 4 arc minute gap. A fixation point at the center of the target was used to maintain foveal fixation. Subjects viewed the stimulus through a 3 mm artificial pupil. Depth of modulation was fixed at 100% (i.e., the stimulus varied from completely on to completely off) and the frequency of the target was adjusted by the experimenter. CFF was calculated as the average of an ascending and descending trial (i.e., frequency was either increased until the target perceptually fused or decreased until flicker was first detected, respectively).

In order to determine sensorimotor ability, reaction time and coincidence anticipation timing were assessed using a linear light array consisting of 120 LEDs (broad band white, λmax  = 460 nm) placed 2.02 centimeters apart on a 3.08 meter long track mounted at a height of 1.68 meters and a distance of 1.22 meters from the subject, as described previously by Renzi et al (2013). Reaction time was measured as the time in milliseconds required for the subject to press a button in response to the illumination of an LED presented at a random location along the linear light array. A total of 80 trials were completed per subject. The first and last ten trials were in place to allow the subject to become familiar with the task and to account for any trials with an error resulting from an extended button press, and were consequently removed, leaving 60 evaluable trials per subject. The latency between trials was varied between 1000 and 3000 milliseconds, to minimize habituation.

Reaction time testing is a well-established method for gauging health and function of visual processing systems in the brain, and as shown by Renzi et al [36], reaction time ability correlates with retinal L and Z levels. The coincidence anticipation task is a less known task that is also used to determine sensorimotor ability by requiring that a subject correctly judges the moment that a moving stimulus arrives at a specified location (the laboratory equivalent to batting, playing tennis, etc). Simulating an object in flight (like a baseball) is accomplished in this task by the same linear light array described above. In the task, the LEDs in the array were illuminated in sequence along the track, timed so that apparent motion occurred, described as the phi phenomenon. Subjects were required to press a button in response to the arrival of the moving light bar (created by lighting individual LEDs in a rapid sequence) at a designated point along the track, as described by Renzi et al [36]. The speed was randomly varied between 5, 10, 15, and 20 miles per hour (mph) for 60 trials, 15 trials for each velocity, with a between-trial latency varying randomly between 1000 and 3000 milliseconds. Several factors contribute to high accuracy on the task: the rate of movement of the light; the accuracy of its representation across the retina (along with accurate smooth pursuit eye movements); the conduction efficiency to the visual cortex and superior colliculus; forward transmission to extrastriate areas, likely V5; transmission along the dorsal (parietal) pathway eventually to the primary motor cortex; neural limb response. A similar pathway is involved in simple reaction time. The entire sequence requires an extremely efficient nervous system because in the fastest speed trials, a motor response must be made prior to conscious awareness of the stimulus.

Response error was calculated as the time in milliseconds of a response made before or after the target, and a trial was designated a “missed” trial if a button press did not occur before the light bar arrived at the end of the track. Trial activation, randomization of LEDs, and button presses were controlled and recorded with customized software and custom-designed electronics.

### Statistical analyses

Results were analyzed with SPSS 17.0. Cross-sectional relationships were described by Pearson product-moment correlations (one-tailed, statistical significance set at *p*<0.05). Changes in dependent variables after supplementation were assessed with paired samples *t*-tests. For most of the analyses, the results are presented for the intervention groups combined. This was done in order to increase statistical power. When analyzed separately, MP density increased by about the same degree in both intervention groups. Since the groups had an equivalent change in L and Z status (as defined by MP density), we analyzed changes in visual processing speed variables across both treatment conditions in order to increase the statistical power of our tests.

## Results

**Table 1** lists the reliability coefficients for measures of MPOD, temporal vision, and sensorimotor ability. Measures of MP were highly reliable (Cronbach's α = 0.93). Good internal consistency (Cronbach's α of 0.80 on average) was also found for measures of reaction time and coincidence anticipation timing error at 10 mph and number of missed trials at 15 mph and 20 mph. Coincidence anticipation timing error at 5 mph, however, had poor internal consistency with a Cronbach's α of 0.51.

**Table 1:** Descriptive statistics (mean ± standard deviation) for each baseline measure and associated reliability estimates (Cronbach's alpha).

	Baseline 1	Baseline 2	N^1^	α
Macular Pigment (30′ eccentricity)	0.36±0.16	0.37±0.17	86	0.93
Critical Flicker Fusion Thresholds (hertz)	26.14±2.95	26.95±2.18	83 2	0.71
Coincidence Anticipation Timing^5^				
Error - 5 MPH	75.21±38.93	69.58±35.65	76 3 ^,^ 4	0.51
Error - 10 MPH	71.79±33.85	67.41±35.67	76 3 ^,^ 4	0.82
Missed - 15 MPH	2.66±2.76	1.48±2.38	76 3 ^,^ 4	0.70
Missed - 20 MPH	6.75±3.82	5.20±3.47	76 3 ^,^ 4	0.80
Reaction Time (ms)	231.01±21.55	225.88±21.85	77 4	0.80

1 Out of 92 subjects, 6 did not complete a second baseline (2 drops and 4 time restrictions during over-enrollment). Total N for measurement reliability is 86.

2 Only one baseline for 3 subjects as a result of equipment maintenance.

3 First baseline visit excluded for 1 subject who had difficulty with the task.

4 Only one baseline for 9 subjects as a result of equipment maintenance.

5 Error is expressed in milliseconds (ms); Missed is expressed in total number of trials.

**Table 2** lists the average baseline correlations with MPOD. Subjects with higher macular pigment had higher CFF thresholds, less error at 5 mph and fewer missed trials at 15 mph for the coincidence anticipation timing task. Error at 10 mph, missed trials at 20 mph (the most perceptually difficult speed, with perfect anticipation below threshold for most people), and reaction time were not significantly related to MPOD.

**Table 2:** Baseline correlations between macular pigment and measures of temporal vision and visual motor reaction time (N = 92).

	Macular Pigment (30′ eccentricity)	
	*r*-value	*p*-value
Critical Flicker Fusion Thresholds	0.34	<0.01
Coincidence Anticipation Timing		
Error - 5 MPH	−0.28	<0.01
Error - 10 MPH	−0.11	0.15
Missed - 15 MPH	−0.26	<0.01
Missed - 20 MPH	−0.07	0.26
Reaction Time	−0.07	0.25

Analysis of intervention data indicated significant increases in MPOD at 30 minutes retinal eccentricity after four months of supplementation (see Table 3). Macular pigment significantly decreased at 30 minutes eccentricity for subjects in the placebo group (*p* = 0.03). Changes in CFF and reaction time for subjects in either an active treatment group or the placebo group are listed in **Table 3**. Changes in CFF in the positive direction indicate improvement (e.g., higher flicker frequency required for fusion is indicative of faster processing speed). Changes in reaction time, coincidence anticipation error, and missed trials in the negative direction indicate improvement (e.g, faster reaction time, less coincidence anticipation error, and fewer missed trials are indicative of faster processing speed). CFF thresholds significantly increased for subjects in the treatment groups (N = 54, *t = *2.53, *p* = 0.01), whereas no significant changes were found for subjects in the placebo condition (N = 10, *t* = 0.19, *p* = 0.86). Reaction time significantly decreased for the treatment group (*t* = 3.60, *p*<0.01) and subjects in the placebo group did not have significant changes in reaction time (*t* = 0.12, *p* = 0.91). Coincidence anticipation error did not significantly change for the treatment or placebo group (see Table 3). Number of missed trials for coincidence anticipation timing tasks, however, significantly decreased for the treatment group (15 MPH: *t* = 2.54, *p* = 0.01; 20 MPH: *t* = 2.78, *p*<0.01). No significant changes were found for the placebo group (15 MPH: *t* = 1.84, *p* = 0.10; 20 MPH: *t* = 0.75, *p* = 0.47).

**Table 3:** Changes in macular pigment, critical flicker fusion thresholds, and reaction time (mean +/− SD) after four months of supplementation for subjects either in an active treatment group (N = 54) or the placebo group (N = 10).

Variable	Group	Baseline	Final	Change	t-value	p-value
Macular Pigment (30′ eccentricity)	Zeaxanthin (N = 29)	0.40±0.15	0.49±0.16	+0.09	4.44	<0.01
	Multi (N = 25)	0.33±0.15	0.42±0.16	+0.09	4.67	<0.01
	Placebo	0.42±0.18	0.39±0.16	−0.03	2.69	0.03
Critical Flicker Fusion Thresholds (hertz)	Treatment	26.60±2.04	27.34±2.25	+0.74	2.53	0.01
	Placebo	28.50±3.76	28.61±2.11	+0.11	0.19	0.86
Coincidence Anticipation Timing^1^						
Error - 5 MPH	Treatment	68.47±27.77	73.39±37.79	+4.92	0.99	0.33
	Placebo	77.71±47.12	83.94±47.43	+6.23	0.73	0.49
Error - 10 MPH	Treatment	70.48±32.85	69.42±33.82	−1.06	0.32	0.74
	Placebo	75.51±32.81	69.70±44.63	−5.81	0.76	0.47
Missed - 15 MPH	Treatment	2.29±2.46	1.59±2.42	−0.70	2.54	0.01
	Placebo	2.00±2.37	1.20±1.55	−0.80	1.84	0.10
Missed - 20 MPH	Treatment	6.63±3.67	5.52±3.75	−1.11	2.78	<0.01
	Placebo	5.35±2.86	4.70±4.30	−0.65	0.75	0.47
Reaction Time (ms)	Treatment	229.94±23.30	223.36±21.64	−6.58	3.60	<0.01
	Placebo	219.63±14.17	220.07±20.44	+0.44	0.12	0.91

1 Error is expressed in milliseconds (ms); Missed is expressed in total number of trials.

## Discussion

The macular carotenoids L and Z appear to influence many aspects of central nervous system function. These effects extend from optical filtering within the neural retina to improving the efficiency of well-established processing streams in the brain and motor systems. MPOD, measured as a surrogate for L and Z levels throughout both the retinal and post-retinal visual system, has been significantly related to fixed and variable reaction time, coincidence anticipation errors (estimating the arrival of a stimulus at a target location moving at varying velocity), and balance ability [36]. Reduced processing speed is a central feature of cognitive decline, and current data suggest that higher MPOD is related to preservation of cognitive function [26], [32]–[33].

The idea that L and Z may directly improve neural function within the brain was formally stated as the Neural Efficiency Hypothesis [30]. The current study was a test of this hypothesis. The visual stimuli that were used in this experiment were specifically designed to test central measures of visual processing. Hence, the mechanisms underlying the behavioral responses likely also reflected functional properties of the brain as opposed to only optical or neural properties of the eye itself, such as improved function in disabling glare conditions [37] or reduced scotopic noise [38]. Indeed, CFF has been associated with cognitive performance, which suggests that relations with L and Z may reflect a common mechanism related to neural processing speed [39].

In the first phase of the study, we evaluated (on a large sample, n = 92) the reliability of our dependent measures. All of the variables that we assessed were highly reliable (average Cronbach's alpha was 0.78), except coincidence anticipation timing at the lowest speed. These variables were then used in the first phase on the larger sample to examine cross-sectional relationships. In general we found moderate, but statistically significant, correlations between most of our functional measures and MPOD.

The second phase of the study was our intervention. The primary outcome variable was increasing MPOD. Many recent studies have found that increases in MPOD tend to be quite muted, and those studies tend to report a significant number of retinal non-responders [40]. For example, Nolan et al. [41] supplemented 12 mg of L and Z for one year in a relatively large sample of healthy volunteers. Their final change after a year of supplementing was 0.10, but that change did not occur until after about 9 months of supplementation. This outcome is similar to many recent large trials. The increasing rise in digestive issues (e.g., celiac' s disease) and the general lack of deficiency due to greater awareness of the beneficial health effects of supplementing L and Z may mean that it is harder to achieve significant increases in MPOD, especially over a shorter time period. Nonetheless, both of our intervention groups did have significant increases in MPOD as measured at the central location (30 minutes) and using the standard one-degree measure. The overall increase was similar to Nolan et al. [41]; see Table 3. Our increased response was likely due to the larger doses that we used.

In the intervention phase of the study, we tested visual processing speeds in a group of younger healthy individuals to determine if four months of supplementation (with the accompanying improvement in L and Z status) would result in change in our dependent measures. The ability to change dynamic processing in younger individuals is difficult for a variety of reasons. First, our variables (CFF, reaction time, and coincidence anticipation errors) were designed to reflect dynamic output. Static variables are easier to measure because they do not tend to vary with the changing state of the individual. For instance, some known correlates of CFF thresholds are: blood pressure; vascular velocity within the retina; pharmaceutical use (amphetamines, barbituates, etc); smoking and alcohol use; caffeine use; age; pupil size; time of day; intelligence; dementia; mild traumatic brain injury; word decoding ability; eye disease (glaucoma, AMD); and so on [42]–[43]. Simple reaction time and visual motor responses are related to a similar set of variables – in essence, variables that influence the dynamic state of the brain. This study tested whether L and Z had similar effects.

In general, the outcomes were consistent with our initial cross-sectional results: a moderate, but significant, effect on some of our timing variables. Supplementation was related to improved CFF thresholds, reaction time, and coincidence anticipation errors at high speeds. The fact that these three specific categories were affected by increases in MPOD is not surprising, for several reasons. First, especially for young subjects, there are likely ceiling effects. Our subjects were young, healthy affluent university students and some of our variables were likely not sufficiently taxing to the nervous system. For example, the 5 mph condition on the coincidence anticipation test is less likely to discriminate performance than the 15 mph condition. Second, temporal vision, like many aspects of vision, is more prognostic at the extremes. Analogously, one does not see the same magnitude of individual variations for young people when testing at low as opposed to high spatial frequencies. In the temporal domain, it tends to be the fastest and highest frequency that the system is capable of where the highest variation can be seen across young subjects. Finally, this sample was highly homogenous and there were few subjects that had low MPOD (only three lower than 0.20). Many neural effects are most apparent in a deficiency state; enhancing is always more difficult than correcting.

In sum, these results are consistent with a role of L and Z in visual processing speed and visual motor behavior. The mechanism responsible for such changes is likely different from common pharmaceutical approaches (like caffeine) and may involve more fundamental changes to cellular connectivity. For example, recent preliminary data has shown that lutein may influence the differentiation of pluripotent neural stem cells [44] (Kuchan et al., 2013). Carotenoids may also influence the production of connexin proteins promoting intercellular communication [45]–[46].(Stahl et al., 1997; Bertram, 1999). The fact that we could produce measurable changes in young healthy individuals is promising given the high therapeutic and practical relevance of a faster more efficient nervous system.
